# Evaluation of Endoscopic Ultrasound Image Quality Is Necessary in Endosonographic Assessment of Early Gastric Cancer Invasion Depth

**DOI:** 10.1155/2012/194530

**Published:** 2012-09-16

**Authors:** Shunsuke Yamamoto, Tsutomu Nishida, Motohiko Kato, Takuya Inoue, Yoshito Hayashi, Jumpei Kondo, Tomofumi Akasaka, Takuya Yamada, Shinichiro Shinzaki, Hideki Iijima, Masahiko Tsujii, Tetsuo Takehara

**Affiliations:** ^1^Department of Gastroenterology and Hepatology, Osaka University Graduate School of Medicine, 2-2, Yamadaoka, Suita, Osaka 5650871, Japan; ^2^Department of Gastroenterology, Osaka Rosai Hospital, 1179-3, Nagasonecho, Kita-ku, Sakai, Osaka 5918025, Japan

## Abstract

We evaluated whether endoscopic ultrasonography (EUS) image quality affects the accuracy of diagnosing the vertical invasion depth of early gastric cancer (EGC). A total of 75 lesions in 75 patients suspected of having EGC were enrolled. All patients underwent EUS examination. Findings of EUS were compared with histopathologic results. We evaluated the effect of the following clinicopathologic factors: location, diameter, surface pattern, concomitant ulceration, histology type, and EUS image quality score. EUS image quality was scored based on detection repeatability, appropriate probe placement, and clarity of the five gastric wall layers including the lesion. Sixty-three lesions (84%) were pathologically mucosal and 12 lesions (16%) were submucosal cancer. Overall accuracy was 82.7%. Significantly more lesions in the upper and middle portions of the stomach were incorrectly diagnosed than in the lower portion (*P* = 0.0019). Lesion diameter was significantly larger among incorrectly diagnosed lesions (*P* = 0.0257). Low-quality images were significantly more often associated with incorrectly diagnosed lesions than with correctly diagnosed lesions (*P* = 0.0001). Multivariate analysis revealed that EUS image quality was associated with EUS staging accuracy (odds ratio, 21.8; 95% confidence interval, 4.5–137.6). Low-quality EUS images led to an incorrect diagnosis of invasion depth of EGC, independent of tumor location or size.

## 1. Introduction

Pretherapeutic diagnosis based on the invasion depth of early gastric cancer (EGC) has become increasingly important with the development of endoscopic submucosal dissection (ESD) techniques. Although endoscopic ultrasonography (EUS) is considered useful for diagnosing the vertical cancer invasion depth, there are contradictory reports on the role of EUS in diagnosing EGC [1, 2]. Clinicopathologic factors of tumors, including the size [3, 4] and location of the lesion [3, 5, 6], gross morphologic type [5, 6], concomitant ulceration [4, 7], and histologic type [3], are reported to affect the diagnostic performance of EUS. Factors influencing the accuracy of EUS-based diagnosis, however, differ among studies and a consensus has not yet been reached [2, 3, 6, 8]. In addition, endoscopic skill or practical technical difficulties also influence the ability to make an accurate diagnosis. We considered that practical technical difficulties of achieving adequate EUS images such as probe placement and scanning at a constant distance from the lesion might influence diagnostic performance. It is difficult to evaluate technical problems or skill quantitatively, but we hypothesized that the quality of the EUS images is important to eliminate the factors of technical problems or skill that may affect the diagnosis. Measuring the depth of EGC using poor-quality EUS images might lead to incorrect results, irrespective of tumor-related factors such as tumor location and size. We hypothesize that the EUS image quality affects the diagnostic accuracy of EUS regarding the EGC invasion depth. To the best of our knowledge, there are no reports on this aspect. The aim of the present study was to elucidate the influence of the quality of EUS images on the diagnostic accuracy of EUS for assessing vertical invasion of EGC.

## 2. Patients and Methods

### 2.1. Patients and Lesions

 A total of 77 lesions in 77 consecutive patients suspected for EGC endoscopically from April 2007 to July 2008 were retrospectively investigated. We excluded two lesions because one was considered advanced cancer before analysis and the other was in a reconstructed gastric tube following esophageal cancer. Thus, a total of 75 lesions were enrolled and analyzed in this study. All patients underwent EUS examination with an endosonography catheter probe before treatment at Osaka University Hospital, Osaka, Japan. The patients were then treated by ESD or gastrectomy in our hospital based on the endoscopic diagnosis. ESD was basically indicated according to the criteria of node-negative EGC established by Gotoda et al. [9] and gastrectomy was indicated if the EGC was more advanced than allowed by the ESD criteria. All the patients provided written informed consent before undergoing examination and treatment. 

### 2.2. EUS Diagnosis

 We used a 2.5 mm diameter miniature ultrasonic probe UM-2R or 3R (Olympus, Tokyo, Japan) in the study. The UM-2R or UM-3R ultrasonic probe incorporated a radial scanning system with a frequency of 12 MHz or 20 MHz, respectively. These were connected to an endoscopic ultrasonic observation unit (EU-M2000; Olympus, Tokyo, Japan). Nonaerated water was instilled to improve transmission of the ultrasound beam. EUS examinations were performed by 4 investigators (S.Y, M.K, Y.H, and T.K) whose years of EUS experience were 3, 3, 2, and 5 years, respectively. We classified the findings of the EUS images of tumor lesions into EUS-M and EUS-SM according to the method by Yanai et al. and Mouri et al. with some modification [10, 11]. EUS-M was defined to include pathologic, minute (500 *μ*m) invasive cancer (sm1) in the submucosa because differentiating “sm1” from “m” is very difficult and the therapeutic strategy is very similar. If the lesion was confined within sonographic layers 1 and 2, we considered the lesion as EUS-M. Lesions with obvious irregular narrowing or budding into sonographic layer 3 were defined as EUS-SM. After treatment, we histologically examined specimens that were resected endoscopically or surgically, and compared EUS findings with histologic findings if the pretherapeutic diagnosis was correct. The lesions were defined as sm1 in cases of histologic invasion within 500 *μ*m beyond the mucosa and sm2 in cases of histologic invasion of more than 500 *μ*m. Sensitivity was defined as the proportion of lesions defined as sm2 relative to those defined as EUS-SM. Specificity was defined as the proportion of lesions with less than sm1 relative to those defined as EUS-M. Accuracy was defined as the proportion of the number of true diagnoses divided by the total number of patients.

**Figure 1:**
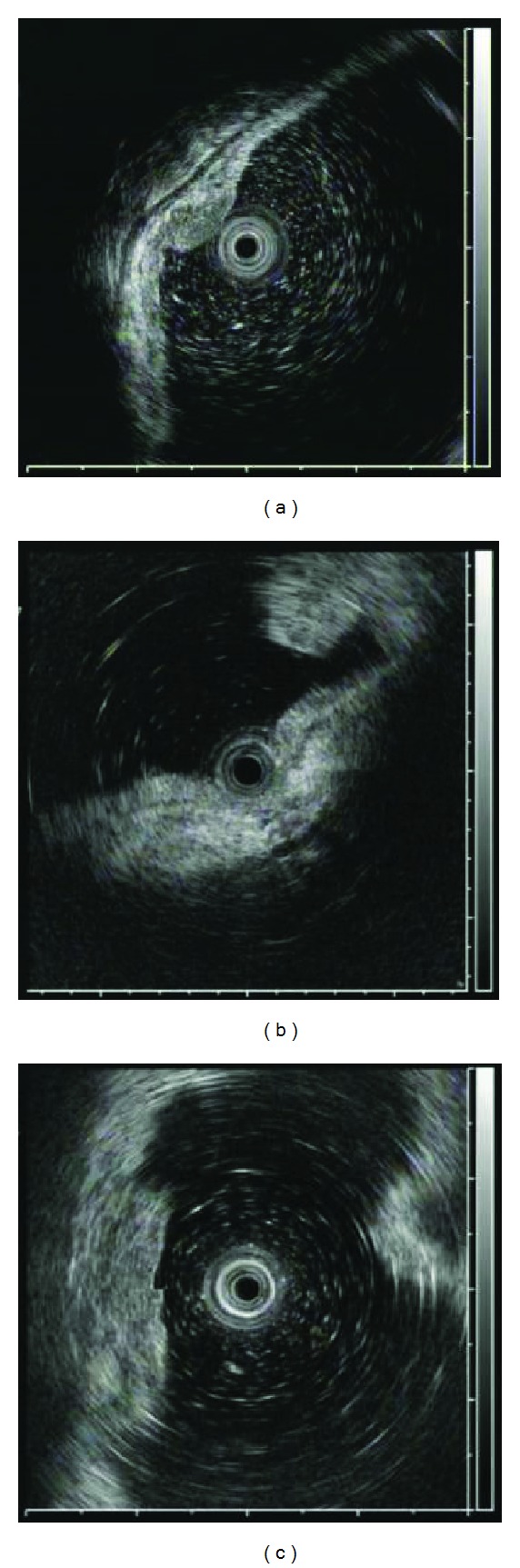
Representative EUS images for evaluating EUS image score. (a) An EUS image fulfilling the three characteristics, including “repeatability of detection,” “appropriate probe placement,” and “clarity of the five layers of the gastric wall including the lesion”; (b) Inappropriate placement of the probe. The EUS probe is pressed on the lesion; (c) the five layers of the gastric wall including the lesion are not clear.

 To determine the factors that influenced the diagnostic accuracy of EUS, we evaluated the following clinical and histologic parameters; location (upper, middle, and lower third of the stomach), tumor size (mm), gross morphologic type (elevated or depressed), concomitant ulceration (endoscopic presence or absence), histologic type (intestinal-type or diffuse-type), and quality of the EUS images. To investigate the influence of the EUS image quality, one of the authors (a physician, S.Y.) retrospectively reviewed and evaluated all EUS images of the lesions based on the following parameters in the blinded manner of pathologic results, and scored them as follows: (1) repeatability of detection (presence [1] or absence [0]), (2) appropriate placement of the probe (ensuring the proper spacing between the probe and the lesion [1]) or impingement of the probe (probe was positioned too close to the lesion; [0]), and (3) clarity of the five layers of the gastric wall including the lesion (clear [1] or unclear [0]). The scores were summed (total ranged from 0 to 3) to calculate the quality of the EUS image of each lesion. The score was stratified as either a low score (scores 0 and 1) or a high score (scores 2 and 3). Typical images of each factor are shown in Figure 1. Finally, multivariate logistic regression analysis was performed to identify the variables among these clinicopathologic factors.

### 2.3. Statistical Analysis

All continuous variables were expressed as mean ± standard deviation (SD). For two-group comparisons, continuous variables were analyzed using Student's *t*-test, and categorical variables using the Fisher's test. Data analysis including multivariate logistic regression analysis was performed with the JMP 8-statistical package (Statistical Analysis Systems Inc, Cary, NC). A *P* value of less than 0.05 was considered statistically significant.

## 3. Results

### 3.1. Patients and Lesions

 A total of 75 lesions were included in this study (62 men and 13 women; mean age: 67 years; range: 41–86 years). ESD was selected to treat 59 lesions and surgery was selected to treat 16 lesions. Analysis of the resected specimens revealed that 63 lesions (84%) were pathologically mucosal and 12 lesions (16%) were submucosal cancer. Location, tumor size, gross morphologic type, concomitant ulceration, histologic type, and EUS image quality of all lesions are shown in Table 1.

### 3.2. EUS Diagnosis

Among the 75 lesions, the overall accuracy of the EUS assessment of the tumor invasion depth was 82.7% (62 of 75 lesions). Sensitivity and specificity were 37.5% (6 of 16 lesions) and 94.9% (56 of 59 lesions), respectively. Among the 13 incorrectly diagnosed lesions, 5.1% (3 of 59 EUS-M lesions) were underdiagnosed and 62.5% (10 of 16 EUS-SM lesions) were overdiagnosed (*P* < 0.0001; Table 2). 

**Figure 2:**
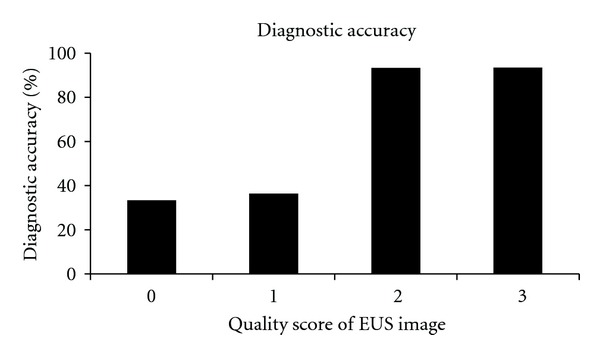
Association between the quality of EUS images and diagnostic accuracy.

The EUS accuracy was not different according to the gross morphologic type, concomitant ulceration, or histologic type of EGC. The EUS accuracy was decreased for lesions in the upper part of the stomach, larger lesions, and lower-quality EUS images (Table 3). The association between the quality of EUS images and the proportions of correct diagnosis are shown in Figure 2. There were five “sm1” lesions in this study. Among them, 3 were diagnosed as EUS-M (correct; image quality score was 3 in all 3 cases), and other 2 were diagnosed as invading over 500 *μ*m by EUS (the score of one case was 1 and that of the other was 2).

 Multivariate analysis using six parameters (location, tumor size, gross morphology type, concomitant ulceration, histology type, and EUS image quality) revealed that EUS image quality was an independent factor with a significant effect (odds ratio, 21.8; 95% confidence interval, 4.5–137.6). 

## 4. Discussion 

 In the present study, the overall accuracy of EUS assessment of tumor depth invasion was 82.7% and the location of the lesion and tumor size were the major factors influencing the diagnostic performance of EUS, similar to previously reported findings [3, 4, 7, 8, 12–14]. Tsuzuki et al. reported that the submucosal layer is relatively thin and tends to have fibrosis and many vessels in the upper third of the stomach, making signs of submucosal invasion difficult to detect and leading to incorrect staging [6]. Our results slightly differed from previous reports, however, in that lesions in the middle third as well as the upper third of the stomach were at high risk for incorrect staging compared to the lower third. We considered the following three explanations. First, in the upper and middle third of the stomach, adequate filling with water is often difficult and may result in an unclear EUS image. Second, progression of atrophic or metaplastic gastritis surrounding the tumor might affect tumor appearance in EUS images. Third, it is technically difficult to precisely horizontally place EUS probes in lesions in the inferior wall in the body or in the lesser curvature in the angle of the stomach, which results in unclear or inaccurate EUS images. In this study, overstaging was the major cause of our incorrectly staged lesions. Yanai et al. reported that EUS tended to result in overstaging while observation under white light endoscopy tended to result in understaging [15, 16]. In addition, incorrect staging is reported to be caused by inflammation associated with ulcers, benign cystic glands in the submucosal layer, and attenuation of the high-frequency ultrasound beam [16].

 To clarify the factors influencing inaccurate diagnosis by EUS in this study, we investigated the EUS image quality in addition to the well-known clinicopathologic factors assessed to determine the invasion depth of EGC using a high-frequency EUS probe. Although it was difficult to evaluate technical problems or skills quantitatively and to eliminate the subjective view of the operator, which may strongly affect the EUS diagnosis, we considered that we were able to evaluate the impact of technical problems or skills by assessing the quality of EUS images. We found that the EUS image quality was an independent factor that affected diagnosis accuracy. To the best of our knowledge, this is the first study to evaluate EUS image quality. We chose the following three factors to evaluate EUS image quality: (1) repeatability of detection, (2) appropriate probe placement, and (3) the clarity of the five layers of the gastric wall including the lesion, because we considered that these factors could be not difficultly and objectively assessed. Based on our results, EUS images with a score of 1 or lower may be insufficient for making an accurate diagnosis of the invasion depth of EGC. The advantage of using an EUS image score is that the usefulness of each EUS image can be objectively assessed, in addition to factors such as tumor location or size. Among tumors with image quality scores of 3, however, three (6.5%) were incorrectly staged (2 lesions were overstaged and 1 was understaged). A strict and highly structured technique may improve the accuracy [17]. Well-experienced endosonographers might be able to produce endoscopic ultrasound images with better quality and to increase diagnosis accuracy. However, we think that the results by average endoscopists in this study may be rather practical. Whether comprehensive diagnosis can be made with EUS and conventional endoscopy must be confirmed in future studies, and may provide helpful information for the staging [18, 19].

 The present study has several limitations due to the fact that, was a retrospective study. First, to some extent, there was a potential selection bias, but this type of bias may have been minimized by the consecutive patient enrollment. Next, only one investigator judged the EUS score to avoid bias in this study. In the future, evaluation of the EUS scores should be made in concordance with several physicians. Other limitations were the small number of patients and the lack of comparison data with other diagnostic modalities for EGC, especially conventional endoscopy. The importance of using EUS images to evaluate the diagnostic accuracy of invasion depth of EGC must be prospectively confirmed in future studies. 

In conclusion, we elucidated that high-quality EUS images increased the diagnostic accuracy of EGC invasion depth. Thus, lower-quality EUS images may lead to an inaccurate diagnosis and this finding should be taken into account in the evaluation.

## Figures and Tables

**Table 1:** Characteristics of the included lesions.

Characteristics	No. (%)
Location	
Upper third	14 (18.4)
Middle third	32 (42.1)
Lower third	29 (38.2)
Tumor size (mean ± SD, mm)	17.6 ± 11.5
Gross morphologic type	
Depressed	38 (51)
Elevated	37 (49)
Concomitant ulceration	
Present	17 (23)
Absent	58 (77)
Histologic type	
Intestinal	68 (91)
Diffuse	7 (9)
EUS image quality	
0	3 (4)
1	11 (14.7)
2	15 (20)
3	46 (61.3)

**Table 2:** Comparison of EUS and pathologic staging.

	Pathologic m/sm1	Pathologic sm2 or more
EUS-M	56	12
EUS-SM	3	4

**Table 3:** Univariate analysis of clinicopathologic factors for diagnostic accuracy of EUS.

Characteristics		*N* (%) of correct diagnosis	*P* value
Location	Upper, middle, lower	6/14 (42.9), 25/32 (78.1), 28/29 (96.6)	0.0019
Tumor size	Correct/incorrect (mm)	16.2 ± 10.0 (62)/24.0 ± 15.7 (13)	0.0257
Gross morphologic type	Depressed/elevated	32/38 (84.2)/30/37 (81.1)	0.7204
Concomitant ulceration	Present/absent	13/17 (76.5)/49/58 (84.5)	0.4428
Histologic type	Intestinal/diffuse	57/68 (83.8)/5/7 (71.4)	0.4094
Quality of EUS images	Low (0,1)/high (2,3)	5/14 (35.7)/57/61 (93.4)	<0.0001

## References

[1] Choi J, Kim SG, Im JP, Kim JS, Jung HC, Song IS (2010). Comparison of endoscopic ultrasonography and conventional endoscopy for prediction of depth of tumor invasion in early gastric cancer. *Endoscopy*.

[2] Kwee RM, Kwee TC (2008). The accuracy of endoscopic ultrasonography in differentiating mucosal from deeper gastric cancer. *American Journal of Gastroenterology*.

[3] Kim JH, Song KS, Youn YH (2007). Clinicopathologic factors influence accurate endosonographic assessment for early gastric cancer. *Gastrointestinal Endoscopy*.

[4] Okada K, Fujisaki J, Kasuga A (2011). Endoscopic ultrasonography is valuable for identifying early gastric cancers meeting expanded-indication criteria for endoscopic submucosal dissection. *Surgical Endoscopy and Other Interventional Techniques*.

[5] Park JM, Ahn CW, Yi X (2011). Efficacy of endoscopic ultrasonography for prediction of tumor depth in gastric cancer. *Journal of Gastric Cancer*.

[6] Tsuzuki T, Okada H, Kawahara Y (2011). Usefulness and problems of endoscopic ultrasonography in prediction of the depth of tumor invasion in early gastric cancer. *Acta Medica Okayama*.

[7] Akashi K, Yanai H, Nishikawa J (2006). Ulcerous change decreases the accuracy of endoscopic ultrasonography diagnosis for the invasive depth of early gastric cancer. *International Journal of Gastrointestinal Cancer*.

[8] Kim GH, Park DY, Kida M (2010). Accuracy of high-frequency catheter-based endoscopic ultrasonography according to the indications for endoscopic treatment of early gastric cancer. *Journal of Gastroenterology and Hepatology*.

[9] Gotoda T, Yanagisawa A, Sasako M (2000). Incidence of lymph node metastasis from early gastric cancer: estimation with a large number of cases at two large centers. *Gastric Cancer*.

[10] Yanai H, Matsubara Y, Kawano T (2004). Clinical impact of strip biopsy for early gastric cancer. *Gastrointestinal Endoscopy*.

[11] Mouri R, Yoshida S, Tanaka S, Oka S, Yoshihara M, Chayama K (2009). Usefulness of endoscopic ultrasonography in determining the depth of invasion and indication for endoscopic treatment of early gastric cancer. *Journal of Clinical Gastroenterology*.

[12] Choi J, Kim SG, Im JP, Kim JS, Jung HC, Song IS (2010). Is endoscopic ultrasonography indispensable in patients with early gastric cancer prior to endoscopic resection?. *Surgical Endoscopy and Other Interventional Techniques*.

[13] Hizawa K, Iwai K, Esaki M, Matsumoto T, Suekane H, Iida M (2002). Is endoscopic ultrasonography indispensable in assessing the appropriateness of endoscopic resection for gastric cancer?. *Endoscopy*.

[14] Ichikawa T, Kudo M, Matsui S, Okada M, Kitano M (2007). Endoscopic ultrasonography with three miniature probes of different frequency is an accurate diagnostic tool for endoscopic submucosal dissection. *Hepato-Gastroenterology*.

[15] Yanai H, Matsumoto Y, Harada T (1997). Endoscopic ultrasonography and endoscopy for staging depth of invasion in early gastric cancer: a pilot study. *Gastrointestinal Endoscopy*.

[16] Yanai H, Tada M, Karita M, Okita K (1996). Diagnostic utility of 20-megahertz linear endoscopic ultrasonography in early gastric cancer. *Gastrointestinal Endoscopy*.

[17] Matsumoto Y, Yanai H, Tokiyama H, Nishiaki M, Higaki S, Okita K (2000). Endoscopic ultrasonography for diagnosis of submucosal invasion in early gastric cancer. *Journal of Gastroenterology*.

[18] Choi J, Kim SG, Im JP, Kim JS, Jung HC, Song IS (2011). Endoscopic prediction of tumor invasion depth in early gastric cancer. *Gastrointestinal Endoscopy*.

[19] Abe S, Oda I, Shimazu T (2011). Depth-predicting score for differentiated early gastric cancer. *Gastric Cancer*.

